# Dosimetric Risk Factors for Acute Radiation Pneumonitis in Patients With Prior Receipt of Immune Checkpoint Inhibitors

**DOI:** 10.3389/fimmu.2021.828858

**Published:** 2022-01-13

**Authors:** Jianping Bi, Jing Qian, Dongqin Yang, Lu Sun, Shouyu Lin, Ying Li, Xudong Xue, Tingting Nie, Vivek Verma, Guang Han

**Affiliations:** ^1^ Department of Radiation Oncology, Hubei Cancer Hospital, Tongji Medical College, Huazhong University of Science and Technology, Wuhan, China; ^2^ Department of Biostatistics and Epidemiology, University of Massachusetts, Amherst, MA, United States; ^3^ Department of Oncology, The Fifth Hospital of Wuhan, Wuhan, China; ^4^ Department of Radiology, Hubei Cancer Hospital, Tongji Medical College, Huazhong University of Science and Technology, Wuhan, China; ^5^ Department of Radiation Oncology, The University of Texas MD Anderson Cancer Center, Houston, TX, United States

**Keywords:** radiotherapy, immune checkpoint inhibitor, pneumonitis, dosimetry, risk factors

## Abstract

**Purpose:**

Dosimetric parameters (e.g., mean lung dose (MLD), V20, and V5) can predict radiation pneumonitis (RP). Constraints thereof were formulated before the era of combined immune checkpoint inhibitors (ICIs) and radiotherapy, which could amplify the RP risk. Dosimetric predictors of acute RP (aRP) in the context of ICIs are urgently needed because no data exist thus far.

**Methods and Materials:**

All included patients underwent thoracic intensity-modulated radiotherapy, previously received ICIs, and followed-up at least once. Logistic regression models examined predictors of aRP (including *a priori* evaluation of MLD, V20, and V5), and their discriminative capacity was assessed by receiver operating characteristic analysis.

**Results:**

Median follow-up of the 40 patients was 5.3 months. Cancers were lung (80%) or esophageal (20%). ICIs were PD-1 (85%) or PD-L1 (15%) inhibitors (median 4 cycles). Patients underwent definitive (n=19), consolidative (n=14), or palliative (n=7) radiotherapy; the median equivalent dose in 2 Gy fractions (EQD2) was 60 Gy (IQR, 51.8-64 Gy). Grades 1-5 aRP occurred in 25%, 17.5%, 15%, 2.5%, and 5%, respectively. The only variables associated with any-grade aRP were V20 (p=0.014) and MLD (p=0.026), and only V20 with grade ≥2 aRP (p=0.035). Neither the number of prior ICI cycles nor the delivery of concurrent systemic therapy significantly associated with aRP risk. Graphs were constructed showing the incrementally increasing risk of aRP based on V20 and MLD (continuous variables).

**Conclusions:**

This is the first study illustrating that V20 and MLD may impact aRP in the setting of prior ICIs. However, these data should not be extrapolated to patients without pre-radiotherapy receipt of prior ICIs, or to evaluate the risk of chronic pulmonary effects. If these results are validated by larger studies with more homogeneous populations, the commonly accepted V20/MLD dose constraints could require revision if utilized in the setting of ICIs.

## Introduction

Immune checkpoint inhibitors (ICIs) have revolutionized oncologic care throughout the world and now represent the standard of care for many metastatic or locally advanced cancers. Combining ICIs and radiation therapy (RT) represents a major area of ongoing active investigation aimed to promote synergy between modalities in efforts to potentially improve outcomes (1).

However, it is well recognized that ICIs can cause adverse events that may be additive with those caused by RT. One such example is pneumonitis, which can be caused independently by ICIs as well as from RT (2–4). Some reports have observed relatively high rates of radiation pneumonitis (RP) in patients with combined ICI and RT, including fatal events (5, 6). However, the safety of combined therapy remains poorly understood owing to 1) the relatively recent adoption of ICIs as well as 2) the rise in RT delivered to suprapalliative doses for metastatic cancers (e.g. for oligometastatic or oligoprogressive disease).

It has long been known that radiation dosimetry is a powerful predictive factor for RP (7), especially the mean lung dose (MLD), the volume of lungs receiving ≥20 Gy (V20) (8), and potentially also the volume of lungs receiving ≥5 Gy (V5) (9). However, those data were not in context of ICIs, which makes their applicability to the ICI setting uncertain.

To our knowledge, there are no dosimetric analyses of MLD, V20, and V5 as possible predictors for acute RP (aRP) in the context of ICI therapy. These analyses are urgently needed because the previously recommended thresholds for these dosimetric parameters (8) may not be applicable to the combined RT-ICI setting and could result in an excess aRP risk. The present study was designed to address this knowledge gap in efforts to question whether the commonly accepted dose constraints for MLD, V20, and V5 may require revision in the future as the combined usage of RT and ICIs continues to expand.

## Methods and Materials

### Patients and Treatment

This investigation was approved by the institutional review and ethics board. We conducted a retrospective review of all patients who received thoracic intensity-modulated radiotherapy (IMRT) at our institution from March 2020 to July 2021. From this institutional dataset, patients who had previously received ICIs were included (concurrent therapy was allowed but not used as an exclusion criterion in order to avoid selection biases). Because the study’s aim was to investigate aRP, patients who did not follow-up (with imaging assessment) were excluded.

Patients with a variety of thoracic cancers were included, so workup and follow-up were individualized, but pre-RT workup always involved chest computed tomography (CT) or positron emission tomography (PET)-CT imaging, and follow-up after RT generally consisted of follow-up chest CT (or PET-CT) one month after RT and every 3 months thereafter.

In efforts to reduce selection biases from the intent of RT, this study included patients who underwent definitive RT, palliative RT (i.e., for symptomatology), or consolidative RT (e.g., for oligoprogression). RT was planned and conducted according to the fundamental principles of each paradigm including with dose constraints put forth by QUANTEC and the AAPM Task Group 101 (8, 10). Image guidance was used for all cases, thrice per week in the first week and twice per week in the remaining weeks.

### Toxicity Assessments

The definition of aRP was based on the Common Terminology Criteria for Adverse Events (CTCAE), version 5.0. All suspected diagnoses of pneumonitis (regardless of grade) were centrally reviewed by a multidisciplinary committee consisting of at least one radiologist, pulmonologist, and oncologist. CT (or PET-CT) imaging was reviewed for each patient, which was then compared to the radiation treatment plan; other causes such as infection or tumor progression had to be ruled out using the appropriate workup.

### Statistical Analysis

This study was specifically designed to (*a priori*) evaluate MLD, V20, and V5 (defined as both lungs minus the planning target volume) as candidate dosimetric parameters for aRP; its goal was not to examine other dose-volume metrics because the three aforementioned parameters represent a “common language” to study RP and have been previously validated and/or widely propagated. Despite the use of a variety of dose-fractionation schemes herein, the aforementioned dosimetric parameters are expressed in 2 Gy equivalent doses (EQD2); based on the linear-quadratic model, MiM software was used to convert the physical dose distribution into EQD2 assuming α/β of 10 and 3 for the target and the normal lung, respectively (11). Logistic regression models were used to assess the association between patient characteristics and the aRP risk. The discriminative capacity of models was assessed by the area under the receiver operating characteristic (ROC) curve (AUC). All data analyses were conducted using R software (version 4.1.0) and used 2-sided tests with p<0.05 indicating statistical significance.

## Results

### Characteristics of Patients

Of 3,276 patients who received thoracic irradiation, 52 patients had previously received ICIs. Of these 52 patients, 12 did not have adequate follow-up in order to evaluate for aRP; therefore, 40 patients were included herein. The median follow-up from thoracic irradiation was 5.3 months [interquartile range (IQR), 3.6-7.7 months].

Salient characteristics of the population are shown in **Table 1**. All patients had lung (80%) or esophageal (20%) cancer, and all patients previously received PD-1 (85%) or PD-L1 (15%) inhibitors. The median number of previous ICI cycles was 4 (interquartile range (IQR), 3-6), and thoracic RT commenced at a median of 26 days (IQR, 18-42) thereafter. During thoracic RT, 15 patients received concurrent systemic therapy (n=8 concurrent anti-PD-1/PD-L1 with chemotherapy, n=4 anti-PD-1/PD-L1 monotherapy, n=3 chemotherapy or targeted agents).

**Table 1:** Patient characteristics.

	No. (%) (N=40)
Median age, years (range)	63 (53-66)
Sex	
Female	6 (15.0)
Male	34 (85.0)
Zubrod performance status	
0-1	37 (92.5)
2	3 (7.5)
Cancer type	
Lung cancer	32 (80.0)
Esophageal cancer	8 (20.0)
Smoking History	
Never	14 (35.0)
Former	9 (22.5)
Current	17 (42.5)
ICI type	
PD-1 inhibitor	34 (85.0)
PD-L1 inhibitor	6 (15.0)
History of COPD	2 (5.0)
Prior TRT	5 (12.5)
Cycles of ICIs before TRT	
1-3	16 (40.0)
4-6	17 (42.5)
7-20	7 (17.5)
Concurrent systemic therapy	
No	25 (62.5)
Yes	15 (37.5)

ICIs, Immune checkpoint inhibitors; PD-1, programmed death 1; PD-L1, programmed death ligand 1; COPD, chronic obstructive pulmonary disease; TRT, thoracic radiotherapy.

**Table 2** displays RT-related characteristics of the population. Nineteen patients received definitive RT, 14 patients underwent consolidative RT for oligoprogression while on ICI therapy, and the remainder (n=7) received palliative RT. The median EQD2 for all patients was 60 Gy (IQR, 51.8-64 Gy). Given the heterogeneity in dose/fractionation schemas, all plans were standardized using EQD2 doses; following this action, the median (IQR) MLD, V20, and V5 were 9.5 Gy (5.7-13.3), 15.5% (9.3-24.7), and 34.3% (20.2-51.4), respectively.

**Table 2:** Thoracic radiotherapy characteristics.

	No. (%) (N=40)
Radiation EQD2 (Gy)^*^	
Median (IQR)	60 (51.8-64)
V20, %^†^	
Median (IQR)	15.5 (9.3-24.7)
V5, %^†^	
Median (IQR)	34.3 (20.2-51.4)
MLD, Gy^†^	
Median (IQR)	9.5 (5.7-13.3)
Radiation treatment type, n (%)	
Curative	19 (47.5)
Consolidative	14 (35.0)
Palliative	7 (17.5)
Median radiation dose, Gy (IQR)	
Curative	60.0 (58.4-64.5)
Consolidative	52.5 (48.5-59.0)
Palliative	45.0 (41.5-55.7)
Median dose/fraction, Gy (IQR)	
Curative	2.1 (2.0-2.2)
Consolidative	2.3 (2.0-5.0)
Palliative	3.0 (2.2-3.0)

EQD2, equivalent dose in 2 Gy fractions; IQR, interquartile range; V20, volume of lung receiving ≥20 Gy; V5, volume of lung receiving ≥5 Gy; MLD, mean lung dose; ICIs, immune checkpoint inhibitors; SBRT, stereotactic body radiation therapy; TRT, thoracic radiotherapy.

^*^Assuming an α/β of 10.

^†^Following conversion of all dose-fractionation schemes to EQD2 based on the LQ model, assuming an α/β of 3.

### Incidence and Characteristics of aRP

At the time of last follow-up, 14 (35%) of patients had not developed aRP. Grade 1 (asymptomatic) RP was detected in 10 (25%) patients. Symptomatic grade ≥2 aRP was present in 16 (40%) patients, of which there were 7 (17.5%), 6 (15%), 1 (2.5%), and 2 (5%) cases of grades 2, 3, 4, and 5 aRP, respectively. **Table 3** displays characteristics of the nine patients who developed grade ≥3 aRP. The median time to aRP was 67 days (IQR, 50-88 days); the most common symptoms included fever (n=12), nonproductive cough (n=10), dyspnea (n=8), and wheezing (n=7). Methylprednisolone was delivered to all patients who developed grade ≥2 aRP.

**Table 3:** Individual characteristics of the 9 patients with grade ≥3 aRP.

Age	PS	Smoking	Type of RT	Prescription dose (Gy) and fractionation	V20 (%)	V5 (%)	MLD (Gy)	Type of ICI	Cycles of ICIs	Prior TRT	Concurrent therapy	Grade of aRP
68	1	Yes	Consolidative	55/20	12.9	20.7	6.9	PD-L1	14	Yes	No	3
71	1	No	Consolidative	56/28	26.2	50.0	11.7	PD-1	5	No	No	3
62	1	Yes	Consolidative	48/16	20.2	32.2	11.9	PD-1	2	No	No	3
51	1	No	Palliative	54/26	23.3	51.2	12.7	PD-1	6	No	Yes	3
67	1	Yes	Curative	64/30	26.6	53.5	16.7	PD-L1	5	No	No	3
66	1	No	Consolidative	55/26	20.3	31.2	9.6	PD-1	8	No	No	3
71	1	Yes	Curative	60/30	21.4	39.6	12.8	PD-1	2	No	No	4
54	1	Yes	Palliative	54/27	29.2	57.4	16.1	PD-1	1	No	Yes	5
66	2	Yes	Palliative	44/20	19.4	54.8	10.5	PD-1	4	No	No	5

aRP, acute radiation pneumonitis; RT, radiotherapy; V20, volume of lung receiving ≥20 Gy; V5, volume of lung receiving ≥5 Gy; MLD, mean lung dose; ICIs, immune checkpoint inhibitors; TRT, thoracic radiotherapy.

**Figure 1:**
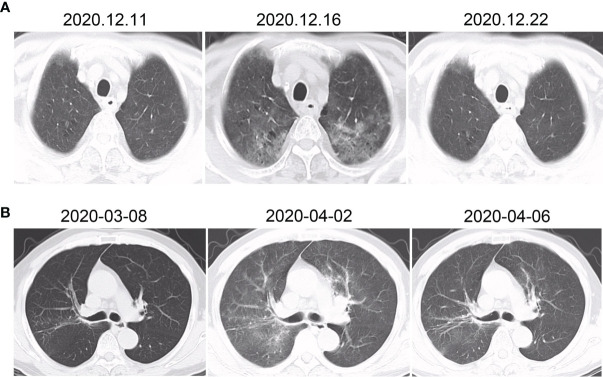
CT changes of the two patients who developed pneumonitis during the course of radiotherapy (both grade 2). The first patient **(A)** developed fever and cough after 23 fractions (50.6 Gy total dose), with no abnormalities initially (left). Five days later, chest CT showed ground-glass changes (center). One week after methylprednisolone commenced, the inflammation had substantially dissipated (right). The second patient **(B)** developed fever after two fractions (9.6 Gy total dose). Initially there were no major findings (left), but patchy infiltrates were soon found in the radiation field (center). After 4 days of methylprednisolone, the findings had significantly reduced.

Of note, two patients developed symptomatic aRP (both grade 2) during the course of RT (the remainder occurred after RT completed) (**Figure 1**). The first had received 50.6 Gy in 23 fractions over 33 days, and the other had received just 9.6 Gy in 2 fractions over 2 days. Both of them were treated with PD-1 inhibitors and had RT interruptions of 10 and 5 days, respectively. Both received methylprednisolone, following which they became asymptomatic and completed the prescribed RT course thereafter.

### Predictors of aRP

**Table 4** illustrates factors associated with the risk of aRP. Regarding any-grade aRP, there were only two associated variables: V20 (odds ratio (OR) 1.117, 95% confidence interval (CI) 1.029-1.232, p=0.014) and MLD (OR 1.205, 95% CI 1.033-1.447, p=0.026). For grade ≥2 aRP, only V20 (OR 1.092, 95% CI 1.011-1.194, p=0.035) was associated. Of note, neither the number of prior ICI cycles nor the delivery of concurrent systemic therapy significantly associated with aRP risk.

**Table 4:** Covariates associated with the development of aRP by univariate regression analysis.

	Any grade aRP			Grade ≥2 aRP		
Risk Factor	OR	95% CI	P Value	OR	95% CI	P Value
Age (continuous)	1.038	0.960-1.129	0.344	1.081	0.994-1.195	0.094
Sex (male vs. female)	4.800	0.805-38.889	0.097	1.400	0.238-11.112	0.719
Smoking (yes vs. no)	1.364	0.366-5.131	0.641	1.088	0.304-3.971	0.897
V20 (continuous)	1.117	1.029-1.232	**0.014**	1.092	1.011-1.194	**0.035**
V5 (continuous)	1.042	0.999-1.091	0.061	1.034	0.994-1.081	0.114
MLD (continuous)	1.205	1.033-1.447	**0.026**	1.145	0.992-1.347	0.077
Prior ICI cycles (continuous)	0.990	0.837-1.189	0.905	0.997	0.833-1.177	0.973
Concurrent systemic therapy (yes vs. no)	0.706	0.184-2.733	0.608	0.636	0.158-2.363	0.506

aRP, acute radiation pneumonitis; OR, odds ratio; CI, confidence interval; V20, volume of lung receiving ≥20 Gy; V5, volume of lung receiving ≥5 Gy; MLD, mean lung dose; ICIs, immune checkpoint inhibitors. The meaning of the bold values is to imply that the P values have significant statistical difference.

**Figure 2:**
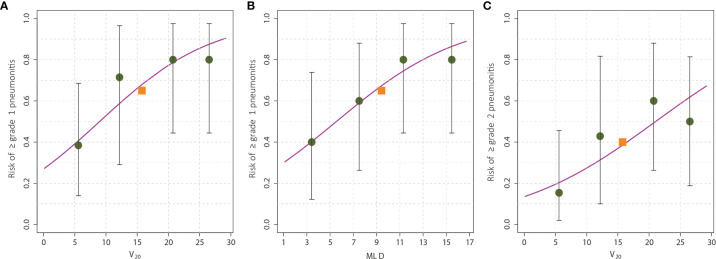
The risk of radiation pneumonitis based on dose-volume parameters. The risk of any-grade radiation pneumonitis was associated with V20 **(A)** and mean lung dose **(B)**, whereas the risk of grade ≥2 pneumonitis was associated with only V20 **(C)**. The observed pneumonitis rate among all patients is denoted by the red square, and that for each quartile of the particular dose-volume parameter is denoted by the black circles. The error bars on the circles represent the exact binomial 95% confidence intervals. Of note, the y-intercept (corresponding to a V20 or MLD of 0) in these graphs is not 0 because immune checkpoint inhibitors carry an independent pneumonitis risk.

ROC analysis is shown in **Figure 2**, and revealed an AUC of 0.762 for the relationship between V20 and any-grade aRP and 0.707 for MLD. The AUC of V20 for grade ≥2 aRP was 0.703. Based on this analysis, graphs were constructed to pictorially present the incrementally increasing risk of aRP based on V20 and MLD as continuous variables (**Figure 2**). According to these models, V20 = 8.98% or MLD=5.55Gy would predict for a 50% risk of ≥ grade 1 pneumonitis (**Figures 2A, B**), and V20 = 21.1% would predict for a 50% risk of ≥ grade 2 pneumonitis (**Figure 2C**). Of note, the y-intercept (corresponding to a V20 or MLD of 0) in these graphs is not 0 because ICIs carry an independent pneumonitis risk.

## Discussion

The use of combined ICIs and RT is rapidly expanding for metastatic (12, 13) and locally advanced cancers (14), but the risk of potentially additive toxicities – especially higher grade events – remains a concern. It is an urgent necessity to report experiences of modifiable variables (e.g., dosimetric parameters) which may alter the risk of toxicities such as aRP. To our knowledge, this is the first study that has observed a relationship between radiation dosimetric variables and aRP in the context of combined RT-ICI therapy. These results undoubtedly require validation, but suggest that the well-recognized dose-volume constraints for thoracic RT may require revision in the future if used in the context of ICI therapy.

The 22.5% rate of grade ≥3 aRP herein is somewhat higher than existing data, which have generally reported figures around 11-15% (15–17). This could be due to two major reasons. First, patients herein received full courses of ICIs prior to RT initiation, whereas in other studies (11–16) most patients did not have as high of a degree of immune system galvanization prior to commencing RT. KEYNOTE-799 (which delivered ICIs before RT) observed a <10% rate of grade ≥3 pneumonitis, but that trial delivered a median of 1 cycle of pembrolizumab/chemotherapy prior to RT (as compared to a median of 4 cycles herein) (18). The higher level of immune activation prior to starting RT in this cohort could have predisposed these patients to develop RP more frequently and with greater severity than patients in the aforementioned publications, but this notion requires further corroboration. Second, a much higher proportion of patients in two other publications (15, 16) received SBRT than those of this investigation, which presumes that irradiated volumes were smaller and more conformally treated than those of the present study. These potential explanations notwithstanding, it is essential for further research to examine whether prior receipt of full-course ICIs are causatively linked to a higher rate of RP, especially because the optimal sequencing of ICIs and RP for metastatic disease remains unknown.

To our knowledge, this is the first investigation which defines dosimetric relationships between lung dose-volume parameters and RP in the context of combined RT and ICIs. Two studies attempted similar goals, but neither could elucidate any such dosimetric factors (15, 16). This is likely related to two major reasons. First, those studies had similarly small sample sizes as this investigation and lower event rates. The publication from MD Anderson Cancer Center observed nine grade ≥3 events in 60 patients (15), and the publication from Emory University documented 19 cases of any-grade RP in 56 patients (16). Conversely, these data are considerably better equipped to detect predictors of RP because it involved a much higher event rate (26 cases of any-grade RP and nine grade ≥3 events in 40 patients). Second, owing to the dearth of available data, all existing studies suffer from heterogeneous patient populations. To that extent, an important strength of this work was that all patients received ICIs targeting the PD-1—PD-L1 axis, whereas the aforementioned publications utilized a mixture of anti-PD-1/PD-L1 agents alone, anti-CTLA-4 compounds alone, and dual immune checkpoint blockade [although it has been suggested that PD-1 and PD-L1 inhibitors have differential rates of pneumonitis (19)].

Owing to similar sample size and heterogeneity concerns as other existing data, as well as the *a priori* nature of dosimetric examination herein, our study also cannot rule out finer differences in other parameters potentially associated with RP that could have gone undetected. That being said, the main message from this study is that V20 and MLD can – and should – continue to be used as important modifiable factors during RT planning. Our study suggests that accepted V20 and MLD constraints (8) may require revision in the future if planning is done in the context of prior ICIs. To this extent, we encourage a more generous utilization of a variety of techniques that could reduce dose exposure to the normal lung, such as deep-inspiration breath hold technique, intensity-modulated or proton therapy, smaller target margins, and high-quality volumetric image guidance.

Importantly, our findings suggest that V5 may not be as robust of a marker with which to predict RP in the context of ICI therapy. This is consistent with its general lack of validation in the non-ICI setting (20), and is potentially reassuring given that full-course ICI therapy prior to RT could activate the immune system to such an extent that even the “low dose bath” could cause RP. However, as mentioned above, finer differences cannot be excluded from any study with smaller sample sizes. Additionally, one case of grade 2 aRP during the RT course herein occurred after just 9.6 Gy in 2 fractions. Even though this patient received a high fractional dose (which in itself could have been the cause of aRP), it suggests that there can indeed be cases of RP at lower doses than would be predicted in the non-ICI setting.

Limitations of this investigation must be contextualized with the fact that the available data on this topic are very scant at the present time. First, as mentioned above, all known data aiming to address dosimetric predictors of RP in the context of ICIs (15–17) are retrospective, with small sample sizes, and short follow-up; along with heterogeneous populations, treatment paradigms, and workup/follow-up. For this reason, this study is not equipped to examine whether concurrent RT & systemic therapy after prior ICIs increases the RP risk over prior ICI therapy alone. Similarly, the contribution of fractional dose (i.e., conventional vs. various degrees of hypofractionation) to RP also cannot be ascertained. It is also unknown whether a greater number of ICI cycles prior to RT increases the risk of RP. Therefore, as the use of combined ICIs and RT increases over time, validating this study and those publications with larger sample sizes is essential. Second, this study should not be extrapolated to situations that do not involve prior ICI therapy. As mentioned above, receipt of full-course prior ICIs could pose a very different RP-related risk than up-front concurrent therapy, RT followed by ICIs, or a short course of ICIs followed by RT. Third, this study only aimed to examine acute RP and should not be used to estimate chronic RP or pulmonary fibrosis. It is possible that this study underestimates the rate of overall RP for this reason, along with the fact that death is a competing risk for RP in this population. Lastly, not all variables can be input into the logistic regression model, especially those with small subgroup sample sizes. We also did not evaluate several other candidate variables potentially associated with RP such as baseline pulmonary function, prior pneumonitis or other adverse events from pre-RT ICI therapy, and size of the planning target volume.

## Conclusions

It is an urgent necessity to report experiences of modifiable variables (e.g., dosimetric parameters) which may alter the risk of toxicities such as aRP. To our knowledge, this is the first investigation that has observed a relationship between radiation dosimetric variables and aRP in the context of combined RT-ICI therapy. We demonstrate that the lung V20 and MLD was independently associated with any-grade RP, and the V20 was associated with grade ≥2 aRP. This study is only applicable for aRP (not chronic RP or pulmonary fibrosis) and in the setting of prior full-course ICI therapy. These results require validation from studies with larger and more homogeneous populations.

## Data Availability Statement

The original contributions presented in the study are included in the article/supplementary material. Further inquiries can be directed to the corresponding authors.

## Ethics Statement

The studies involving human participants were reviewed and approved by the institutional ethics board of Hubei Cancer Hospital of Huazhong University of Science and Technology in Wuhan, China. The patients/participants provided their written informed consent to participate in this study.

## Author Contributions

JB, GH, and VV performed the literature search and study design. JB, JQ, DY, LS, SL, YL, XX, and TN collected the data, processed statistical data, performed and data interpretation. JB, JQ, GH, and VV drafted the manuscript. GH and VV revised the final manuscript. All authors read and approved the final manuscript.

## Funding

This work was supported by National Cancer Center Climbing Foundation (No. NCC201917B03) and Hubei Provincial Health Commission (No. ZY2021M008). JQ is supported by University of Massachusetts Amherst.

## Conflict of Interest

The authors declare that the research was conducted in the absence of any commercial or financial relationships that could be construed as a potential conflict of interest.

## Publisher’s Note

All claims expressed in this article are solely those of the authors and do not necessarily represent those of their affiliated organizations, or those of the publisher, the editors and the reviewers. Any product that may be evaluated in this article, or claim that may be made by its manufacturer, is not guaranteed or endorsed by the publisher.
